# Proximal Limb Weakness in a Patient with Celiac Disease: Copper Deficiency, Gluten Sensitivity, or Both as the Underlying Cause?

**DOI:** 10.1155/2016/5415949

**Published:** 2016-11-22

**Authors:** J. David Avila, David Lacomis

**Affiliations:** ^1^Department of Neurology, Geisinger Medical Center, Danville, PA, USA; ^2^Department of Neurology, University of Pittsburgh Medical Center, Pittsburgh, PA, USA; ^3^Department of Pathology, University of Pittsburgh Medical Center, Pittsburgh, PA, USA

## Abstract

Celiac disease has been associated with several neurologic disorders which may result from micronutrient deficiencies, coexisting autoimmune conditions, or gluten sensitivity. Copper deficiency can produce multiple neurologic manifestations. Myeloneuropathy is the most common neurologic syndrome and it is often irreversible, despite copper replacement. We report the case of a 55-year-old man who presented with progressive proximal limb weakness and weight loss in the setting of untreated celiac disease without gastrointestinal symptoms. He had anemia, neutropenia, and severe hypocupremia. The pattern of weakness raised the suspicion that there was an underlying myopathy, although this was not confirmed by electrodiagnostic studies. Weakness and hematologic abnormalities resolved completely within 1 month of total parenteral nutrition with copper supplementation and a gluten-free diet. Myopathy can rarely occur in patients with celiac disease, but the mechanism is unclear. Pure proximal limb weakness has not been previously reported in copper deficiency. We propose that this may represent a novel manifestation of hypocupremia and recommend considering copper deficiency and gluten sensitivity in patients presenting with proximal limb weakness.

## 1. Introduction

Celiac disease (CD) is a systemic autoimmune condition triggered by dietary gluten in genetically susceptible individuals. It has a prevalence of 0.6 to 1% worldwide [1]. Although historically considered a gastrointestinal disorder, approximately half of patients present with extraintestinal symptoms [1]. Several neurologic disorders have been associated with CD [2–4]. Whether they result from micronutrient deficiencies, coexisting autoimmune disorders, or gluten sensitivity alone is not entirely clear [5].

Copper deficiency may occur in the setting of zinc poisoning, gastric bypass surgery, and malabsorption syndromes. It can manifest with multiple hematologic and neurologic abnormalities. Anemia and neutropenia are the most common hematologic derangements [6]. Among the neurologic manifestations myeloneuropathy and ataxic myelopathy, similar to subacute combined degeneration, have been well described [7]. Other less typical presentations include motor neuron disease, optic neuropathy, and central nervous system demyelination [8–10]. To our knowledge, pure proximal limb weakness has not been previously reported.

Herein, we describe a patient with copper deficiency and CD who presented with symmetric proximal limb weakness and had complete resolution of symptoms with copper replacement and gluten-free diet.

## 2. Case Report

A 55-year-old man with a previous history of peripheral vascular disease, CD, and hypothyroidism presented with 4 months of bilateral leg weakness and weight loss. He was a retired police officer and had been physically very strong in the past. CD was diagnosed elsewhere 5 years earlier. The details of his initial evaluation were not available. The disease was thought to be inactive and the patient was on an unrestricted diet.

The symptoms were slowly progressive. He had difficulty rising from a chair and going up stairs. He denied eyelid ptosis, diplopia, dysarthria, dysphagia, dyspnea, myalgia, fasciculations, muscle atrophy, numbness, paresthesia, and bladder or bowel problems. He had unintentional weight loss of 30 Lb. There was no loss of appetite, decreased food intake, diarrhea, or steatorrhea. Examination demonstrated 4/5 strength in the deltoids and iliopsoas bilaterally (Medical Research Council scale). There was no weakness of intermediate and distal limb muscles. Cranial nerves, reflexes, sensation, coordination, and gait were normal.

He had a 60-pack-year history of smoking and the initial investigations were focused on a possible malignancy. Laboratory workup was remarkable for macrocytic anemia and neutropenia (Table 1). Peripheral blood smear and flow cytometry showed no evidence of leukemia or lymphoma. Bone marrow biopsy demonstrated hypocellularity with no other abnormalities. Computed tomography of the chest, abdomen, and pelvis was normal. Acetylcholine receptor and voltage-gated calcium channel antibodies were negative. Serum creatine kinase and thyroid function tests were also normal. Electrodiagnostic studies (EDX) were performed per our myopathy protocol, which includes one motor and one sensory nerve conduction study (NCS) from an arm and a leg (Ulnar, tibial, and sural NCS in this case), and needle examination of multiple proximal and distal arm and leg muscles as well as a thoracic paraspinal muscle. Additionally, we performed 3 Hz repetitive nerve stimulation of the ulnar, accessory, and facial nerves. There were no abnormalities found on EDX. Further testing revealed severe copper deficiency [6 *μ*g/dL (60–190)] and mild B_12_ deficiency [193 pg/mL (211–911)], as well as positive anti-gliadin and anti-transglutaminase antibodies. Zinc and vitamins D and E were normal (Table 1).

**Figure 1:**
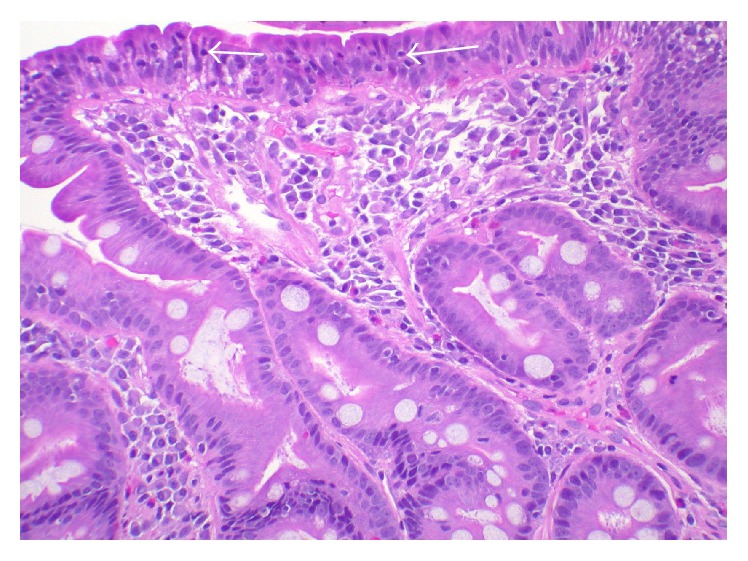
Duodenal biopsy specimen showing villous blunting and intraepithelial lymphocytosis (white arrows). There is also chronic inflammation in the lamina propria and crypt hyperplasia consistent with gluten sensitive enteropathy (hematoxylin and eosin stain).

The patient underwent endoscopy and duodenal biopsy that showed moderate villous blunting, intraepithelial lymphocytosis, chronic inflammation of the lamina propria, and crypt hyperplasia. The histologic findings, in conjunction with the elevated anti-transglutaminase antibodies, confirmed the diagnosis of active gluten sensitive enteropathy or CD (Figure 1). He was placed on a strict gluten-free diet and total parenteral nutrition (TPN) with micronutrient supplementation. The daily dose of copper was 2 mg. He also had weekly B_12_ intramuscular injections.

After 1 month of TPN, copper and other micronutrients normalized (Table 1). Anemia and neutropenia improved. Weakness resolved completely and the patient regained 10 Lb. TPN was stopped and he remained on a gluten-free diet.

## 3. Discussion

Neurologic manifestations have been reported in 8% of patients with CD [2]. They may be present in individuals with or without enteropathy, as defined by an abnormal small bowel biopsy, and may be the initial presentation of the disease [3]. The most common manifestations are cerebellar ataxia, polyneuropathy, and encephalopathy [3]. Neurologic deficits are attributed to CD or gluten sensitivity when there is no alternative explanation and symptoms improve with a gluten-free diet [3, 4].

Different micronutrient deficiencies may occur as a consequence of malabsorption in CD. Interestingly, micronutrient deficiencies can occur in the absence of gastrointestinal symptoms [11]. Copper deficiency may be present in up to 15% of patients with CD [11]. Myeloneuropathy is the most common neurologic syndrome in this scenario and it is often irreversible [12]. Other nutritional deficiencies include vitamin B_12_, folate, vitamin D, and vitamin E, all of which can lead to neurologic complications.

There are conflicting reports in the literature regarding the cause of neurologic disorders in CD. A study of 68 patients with neurologic dysfunction and CD or anti-gliadin antibody positivity found that the majority (57%) had a discernible cause for the deficits, different from gluten exposure [5]. Coexisting autoimmunity was the most common (19%), followed by malabsorption-induced nutritional deficiency (10%). Most patients deteriorated, despite a gluten-free diet [5]. This study suggested that alternative causes for neurologic dysfunction should be sought in CD and that further therapy may be required, in addition to dietary restriction. On the contrary, a large retrospective study of 562 patients with neurologic dysfunction and CD or nonceliac gluten sensitivity (i.e., no enteropathy) reported that all patients responded to a gluten-free diet, regardless of the severity and type of dysfunction [3]. The authors stated that no alternative etiology for the deficits was found, despite extensive investigations. The details of those investigations, however, were not provided.

Our patient presented with symmetric proximal limb weakness, which is the most common pattern of weakness in myopathies [13]. Even though EDX did not confirm a myopathic process, this was our suspicion based on the distribution of weakness and absence of sensory deficits and physical signs of other pure motor syndromes, such as motor neuron disease and neuromuscular junction disorders. Furthermore, a normal EDX does not exclude a myopathy, as several myopathic disorders may be associated with normal results, particularly the metabolic and endocrine myopathies [14]. We felt that hypocupremia and CD were the likely causes of weakness and, since we planned on treating them, we did not pursue a muscle biopsy that was likely to be of low yield unless the patient failed to respond to treatment. In retrospect, the histopathology might have shown nonspecific evidence of myopathy and support that diagnostic entity, but we still believe it was not clinically necessary.

Myopathy is rare in CD, accounting for only 4% of all neurologic manifestations [3]. It has been described in patients with vitamin D and E deficiencies accompanying CD [15, 16]. Hadjivassiliou et al. proposed that myopathy may represent an extraintestinal manifestation of gluten sensitivity and that it may have an immune mechanism [17]. The authors postulated that transglutaminase type 2, the target of anti-endomysial antibodies (EMA) in CD, may be implicated in the immune-mediated muscle injury, as this enzyme is normally present in the endomysial connective tissue. However, in their study of 13 patients with biopsy-proven myopathy, only 2 had positive EMA [17]. Copper levels were not provided.

CD may also coexist with inflammatory myopathies, as it occurs with other autoimmune disorders [17, 18]. In the same study by Hadjivassiliou et al., 6 of 13 patients had pathologic findings similar to polymyositis. All but one improved with immunosuppressive therapy and gluten-free diet [17]. A Spanish study evaluated the presence of antibodies associated with CD in patients with inflammatory myopathies [18]. In their cohort of 51 patients, 17 (31%) had anti-gliadin IgA antibodies. Interestingly, the strongest association was present in patients with sporadic inclusion-body myositis (sIBM), which is known not to respond to immunosuppressive therapy. There were no patients with anti-tissue transglutaminase or anti-endomysial antibodies. Five patients underwent jejunal biopsy and 3 met criteria for enteropathy or CD. Of those, 2 had sIBM and did not respond to gluten-free diet. The other patient had dermatomyositis and had full resolution of weakness with glucocorticoids and gluten restriction [18].

We cannot definitively determine the cause of weakness in our patient. Even though he had vitamin B_12_ deficiency, we feel this is unlikely to explain the symptoms as the deficiency was relatively mild and it typically produces distal limb weakness and sensory loss, due to neuropathy, myelopathy, or a combination of both. Additionally, the neurology of vitamin B_12_ deficiency has been studied extensively, and myopathy has not been described [19]. We consider copper deficiency a plausible culprit, given the severity of hypocupremia, absence of other nutritional deficiencies known to produce proximal limb weakness (vitamins D and E), hematologic abnormalities, and resolution of symptoms with copper replacement and gluten-free diet. If this is the case, this would represent a novel manifestation of copper deficiency, adding to the spectrum of neurologic dysfunction in hypocupremia. Alternatively, the deficits may be the result of direct gluten sensitivity, malabsorption of another, yet unidentified micronutrient, or a combination of mechanisms.

It is important to emphasize that the patient did not have hyperzincemia. Zinc interferes with the intestinal absorption of copper and increases its sequestration in enterocytes by inducing the expression of metallothioneins, which have higher affinity for copper than zinc [20]. Zinc toxicity is a common precipitant of copper deficiency, and it may result from excessive or prolonged intake of zinc supplements and use of zinc-containing denture fixative [7, 8, 10]. In patients with hypocupremia, a normal zinc level should raise clinical suspicion for an underlying gastrointestinal pathology and trigger investigations for malabsorption syndromes, including CD.

This case report has two main limitations. First, we could not definitely localize the clinical deficits to the muscle or the nervous system. Second, we are unable to prove with certainty that copper deficiency was the cause of weakness, as stated before. However, this is our speculation based on the aforementioned reasons. In the future, assessing copper levels in patients with CD and myopathy may clarify this issue.

We recommend considering copper deficiency and gluten sensitivity in patients presenting with proximal limb weakness, even in the absence of clinically overt enteropathy. Additional evidence is required to determine if this manifestation truly represents a myopathy. Copper replacement, in addition to gluten restriction, may lead to complete resolution of symptoms, as occurred in this case.

## Figures and Tables

**Table 1:** Laboratory findings at diagnosis and after 1 month of TPN.

Parameter	Baseline	After 1 month of TPN	Normal range
RBC (×10^12^/L)	**3.82**	4.42	4.13–5.57
Hemoglobin (g/L)	**12.5**	14.8	12.9–16.9
MCV (fL)	**102.3**	**99.3**	82.6–97.4
WBC (×10^9^/L)	**2.6**	6.7	3.8–10.6
ANC (×10^9^/L)	**0.5**	2.1	1.5–7.7
Platelets (×10^9^/L)	369	293	156–369
Copper (*µ*g/dL)	**6**	93	60–190
Ceruloplasmin (mg/dL)	**3.1**	—	22–58
Vitamin B_12_ (pg/mL)	**193**	811	211–911
Zinc (*µ*g/dL)	0.72	0.78	0.55–1.5
Vitamin D (ng/mL)	32	—	30–50
Vitamin E (*µ*g/mL)	8	—	5.5–17
Anti-gliadin Ab IgA (AU)	**165**	**137**	<20
Anti-gliadin Ab IgG (AU)	**39**	**35**	<20
Anti-TTG IgA (units)	**110**	**64**	<20

TPN: total parenteral nutrition; RBC: red blood cells; MCV: mean corpuscular volume; WBC: white blood cells; ANC: absolute neutrophil count; Ab: antibody; AU: arbitrary units; TTG: tissue transglutaminase.
